# Impact of urbanization on predator and parasitoid insects at multiple spatial scales

**DOI:** 10.1371/journal.pone.0214068

**Published:** 2019-04-03

**Authors:** Daria Corcos, Pierfilippo Cerretti, Valerio Caruso, Maurizio Mei, Matteo Falco, Lorenzo Marini

**Affiliations:** 1 Department of Biology and Biotechnology “Charles Darwin”, Sapienza University of Rome, Rome, Italy; 2 Department of Agronomy, Food, Natural Resources, Animals and the Environment (DAFNAE), University of Padova, Legnaro (Padua), Italy; University of Sydney, AUSTRALIA

## Abstract

Landscapes are becoming increasingly urbanized, causing loss and fragmentation of natural habitats, with potentially negative effects on biodiversity. Insects are among the organisms with the largest diversity in urbanized environments. Here, we sampled predator (Ampulicidae, Sphecidae and Crabronidae) and parasitoid (Tachinidae) flower-visiting insects in 36 sites in the city of Rome (Italy). Although the diversity of herbivorous insects in urban areas mostly depends on the availability of flowering plants and nesting sites, predators and parasitoids generally require a larger number of resources during their life cycle, and are expected to be particularly influenced by urbanization. As flower-visitors can easily move between habitat patches, the effect of urbanization was tested at multiple spatial scales (local, landscape and sub-regional). We found that urbanization influenced predator and parasitoid flower-visitors at all three spatial scales. At the local scale, streets and buildings negatively influenced evenness of predators and species richness and abundance of parasitoids probably acting as dispersal barrier. At the landscape scale, higher percentage of urban decreased predator abundance, while increasing their evenness, suggesting an increase in generalist and highly mobile species. Area and compactness (i.e. Contiguity index) of urban green interactively influenced predator communities, whereas evenness of parasitoids increased with increasing Contiguity index. At the sub-regional scale, species richness and abundance of predators increased with increasing distance from the city center. Compared to previous studies testing the effect of urbanization, we found little variation in species richness, abundance and evenness along our urbanization gradient. The current insect fauna has been probably selected for its tolerance to habitat loss and fragmentation, being the result of the intensive anthropogenic alteration occurred in the area in the last centuries. Conservation strategies aimed at predator and parasitoid flying insects have to take in account variables at multiple spatial-scales, as well as the complementarity of resources across the landscape.

## Introduction

Rapid demographic and economic growth has led to increased urbanization and agricultural intensification [1–3]. Consequently, large areas of natural and semi-natural habitats have been extensively modified to make space for urban, industrial and agricultural areas. Today, half of the world’s population lives in cities [4], and this trend is rapidly increasing with potential negative effect on biodiversity. As the dispersal of species between habitat patches is mediated by the surrounding landscape [5–7], buildings and roads can also constitute barriers to organism movement [4,8]. However, urban areas can provide novel food and nesting resources [4,9], and linear man-made structures can even facilitate the dispersal of some species [10,11]. In cities, semi-natural habitats are largely confined to public parks and private gardens, where most of the green spaces are fragments of highly disturbed habitat [1,2,4,5,9,12–15]. The reduction and fragmentation of remnant habitats can strongly influence communities inhabiting urban areas [5,16], usually with negative effects on biodiversity [2,17] and ecosystem functioning [14,18].

Urban open green areas constitute a suitable habitat for numerous flower-visiting insects [19], as these areas include patches rich in flowering plants, both spontaneous and ornamental [2,13]. Although the effect of urbanization on pollinators, especially bees, has been extensively studied (e.g. [2,18,20–23]), less is known about how other flower-visiting groups behave in urban areas. Deguines et al. [24] suggested that non-hymenopteran flower-visitors are expected to decline in consequence of increasing urbanization, as they usually have a lower affinity with urban areas compared to bees. Similarly, Baldock et al. [19] found that hoverfly abundance was reduced in urban sites, whereas the abundance of bees did not differ among urban and natural sites, and their species richness even increased in more urbanized areas. Among non-bee flower-visitor insects, Sphecids (Hymenoptera: Ampulicidae, Sphecidae and Crabronidae) and tachinids (Diptera: Tachinidae) play an important ecological role, as they provide key ecosystem services, such as pollination and natural control [25,26]. Whereas their adults behave as flower-visitors and feed on nectar and pollen, the larval stage behaves as predator (sphecids) or parasitoid (tachinids) [25,26]. In order to persist in urban environments, these groups need access to a wide range of resources, including food for the adults, prey and host for the larvae, and nesting sites (in the case of sphecids). Habitat fragmentation in urban areas can interfere with the movement of insect species in the landscape, disrupting their ability to locate prey/hosts [27–29]. For this reason, higher trophic levels, such as predators and parasitoids, are expected to be particularly sensitive to change in land-use [5,12,30,31]. However, to our knowledge, there are still no studies testing the effect of urbanization on sphecid and tachinid communities.

Urbanization impact can occur at multiple spatial scales [3,4,7,16,30] and species responses depend on properties of the environment as well as on species traits [15,28,32–35]. In general, the increase in urbanization is expected to negatively influence insect movements [23], and exacerbate spatial mismatch between predators/parasitoids and their resources [4,27]. As urban areas are often dominated by species with generalist feeding habits [5], community evenness can also change along the urbanization gradient. At the local scale, the movement of flower-visiting insects is hindered by the presence of buildings and streets. These elements can act as dispersal barriers, and are perceived in different ways by flying insects (e.g. some species are able to fly over streets, but are not able to outmatch buildings). However, flying insects can easily move between habitat patches [4,10,33], and it has been hypothesized that they should be more influenced by resources at the landscape scale rather than by local scale processes [36]. At the landscape scale, the increase in urban area involves a reduction and fragmentation of the semi-natural habitat where many insects forage and nest. This is expected to reduce the number of resources available for both flower-visitors and their prey, and therefore negatively affect both diversity and abundance of sphecids and tachinids. Also, rural and sub-urban areas surrounding cities can serve as species sources due to the lower impact of urban land use [37] and thus species richness and abundance usually increase with increasing distance from the center of highly urbanized areas [38]. As their diversity depends on the presence of alternative food resources and nesting sites in the landscape [5,12,22], they should be able to persist in urban areas, as long as minimum food and nesting requirements are met [13,20,35]. However, it is still not clear which are the factors driving the diversity of flower-visiting predator and parasitoid insects in urban areas.

In this study, we examined how local, landscape and sub-regional scale variables influenced the diversity of sphecids (predators) and tachinids (parasitiods) in 36 sites in the city of Rome. At the local scale, we expected that buildings and streets will act as dispersal barriers for flying insects and thus negatively influence sphecids and tachinids. However, at the landscape scale, not only the amount of urban, but also area and shape of semi-natural habitat can play an important role in influencing predator and parasitoid insects. We therefore hypothesized that, at the landscape scale, their species richness, abundance and evenness are: i) negatively influenced by percentage of urban, and ii) positively influenced by habitat area and compactness (i.e. Contiguity index). The Contiguity index is a metric of patch boundary configuration, and provides useful information on the compactness of the patch shape [39]. We hypothesized the effect of habitat area will interact with habitat Contiguity index, with a decrease in the effect of habitat area as habitat patches became more compact. At the sub-regional scale, we expected to find a positive effect of the distance from city center, as consequence of the large areas of unmanaged green habitat that can be found outside the city boundaries.

## Materials and methods

### Study area

Rome is the largest and most populated city of Italy, and the 4th most populous city in Europe. Founded in 753 BC, Rome is also one of the oldest continuously occupied cities in Europe, with most urban expansion taking place since 1870 [40]. In 2016, the population was estimated at 3.8 million, with a population density of 2232 persons/km^2^ [41]. The climate is temperate, with rainy winters and warm summers. The city of Rome (i.e. the territory circumscribed by the great motorway ring) measures ca 360 km^2^, where approximately 54% is urban (residential, industrial and commercial areas), 16% is urban green (non-agricultural green areas, both artificial and semi-natural, including historical and archaeological sites, public parks and gardens, grasslands, shrublands and forests), and the remaining 30% is covered in agricultural lands, pastures and waters. In Rome, 67 species of sphecids [42] and 129 of tachinids [42,43] have been previously reported, approximately 18 and 27% of the whole Italian fauna [25,44]. However, these checklists are still largely incomplete.

### Site selection and explanatory variables

**Fig 1:**
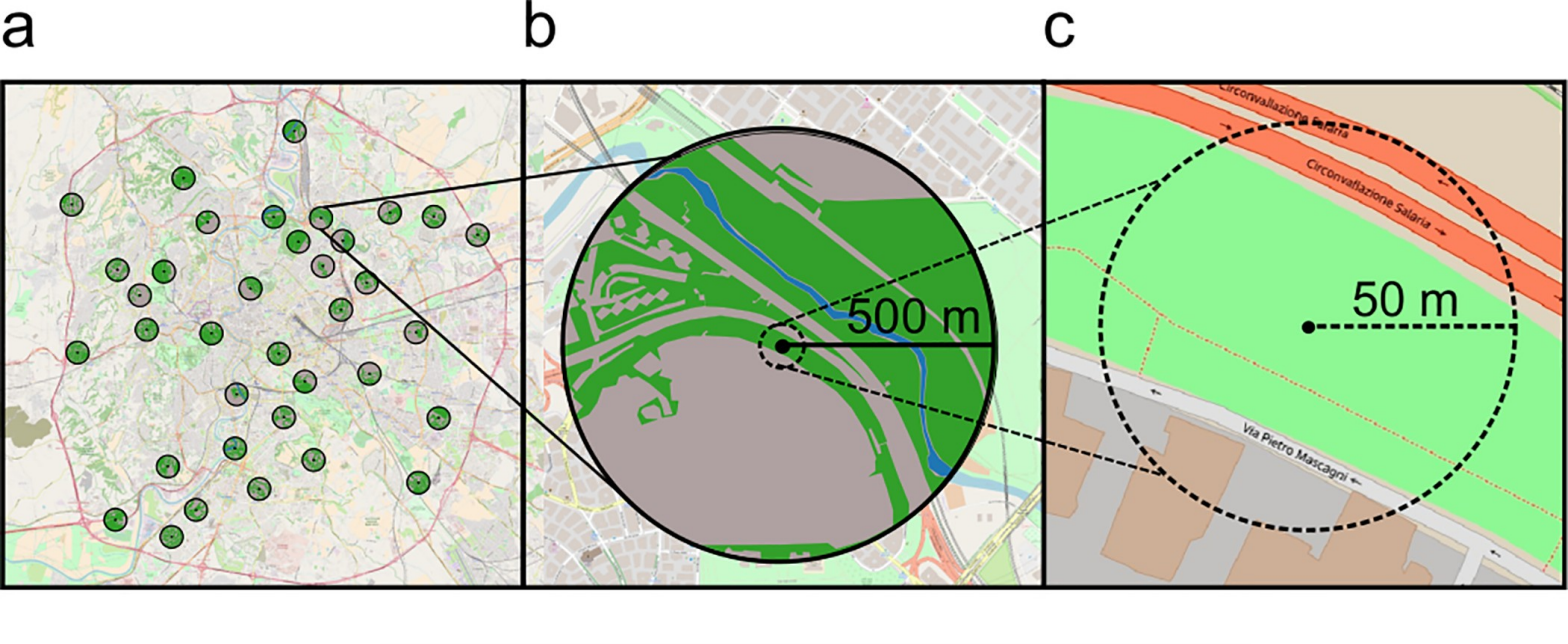
Study area and explanatory variables. a) Spatial distribution of the 36 selected cells in the city of Rome, with indication of the: b) landscape, and c) local scale. The centroid of the cell is the study site where traps were placed. Maps obtained from OpenLayers Plugin, QGIS.

Sampling sites were selected using a map of land use (National Corine Land Cover, 1:10000 scale, 2005–2007, 20 m resolution). We classified as “urban” the Corine classes urban fabric (class 1.1), industrial, commercial and transport units (1.2), and mine, dump and construction sites (1.3). The classes: artificial, non-agricultural vegetated areas (1.4), semi-natural forest (3.1), and semi-natural shrub and/or herbaceous vegetation associations (3.2) where classified as “urban green”. The obtained map was compared with a Google Streets map to check the coherence of the cartographic information, and overlapped with a grid with cells of 1 km x 1 km in QGis Desktop 2.10 [45]. Cells with > 20% agricultural land were eliminated and 36 suitable cells, encompassing a gradient of urban (from 15.3 to 86.1%) and urban green (from 13.9 to 84.7%), were selected (**Fig 1A; S1 Table**). To identify an area of open (i.e. covered in herbaceous vegetation) green habitat close to the centroid of each cell, a field survey was conducted. Sampling sites were patches of open grassland vegetation (hereafter ‘habitat’), covered mainly in spontaneous plants and flowers. The distance between sampling sites ranged from 2 to 26 km. For each sampling point, we measured explanatory variables at the local, landscape and sub-regional scale (**Table 1**). Explanatory variables were not strongly correlated (Pearson’s correlation indices < 0.60; VIF multicollinearity values < 3) (**S2 Table**).

**Table 1:** Description and statistics (mean, median and range) of the explanatory variables considered in the study.

Spatial scale	Explanatory variable	Description	Range	Mean	Median
Local	Distance from street	Distance (m) from the closest street.	3–420	48.8	19.0
	Buildings in 50 m	Building coverage in 360° in a buffer of 50 m from the sampling site.	0–330	88.6	90.0
Landscape (500 m radius buffer)	Percentage of urban	Percentage (%) of residential, industrial, infrastructure, and commercial areas.	15.3–86.1	55.6	58.8
	Habitat area	Total area (ha) of open green habitat.	3.7–53.8	26.5	26.5
	Habitat Contiguity index	Mean contiguity (CONTIG_MN) of open green habitat. It assess the compactness of patch shape and it is based on the spatial contiguity of cells within a patch. It ranges from 0 to 1, with large contiguous patches resulting in larger Contiguity index values.	0.2–0.9	0.5	0.4
Sub-regional	Distance from the city center	Distance (km) to the city centre (Colosseum).	0.7–10.8	6.2	5.9

#### Local scale

At the local scale, we used Google Earth Pro (Google Earth 7.1.5.1557, 2015) to calculate buffers of 50 m radius from each sampling point (**Fig 1C**). We then assessed the coverage in buildings in 360 degrees within the 50 m buffer, and the distance from the sampling point to the closest street. The height of buildings in the study areas was not measured. However, they were always tall enough to be considered as a potential obstacle for flying insects (≥ 4 floors).

#### Landscape scale

At the landscape scale, polygons of urban, urban green (separately open and forest), agricultural land and water were manually digitized in Google Earth Pro (Google Earth 7.1.5.1557, 2015) within a radius of 500 m around each sampling point (**Fig 1B**). Area and Contiguity index (CONTIG_MN) of open green habitat were calculated using FRAGSTAT v4 [46]. The Contiguity index describes the geometric compactness of the habitat type in the landscape, and it is considered a good index of habitat fragmentation [39,47]. Assuming that an habitat patch is defined as an aggregation of pixels, the Contiguity index assesses the spatial arrangement of the pixels within the patch [39]. The metric ranges from 0 to 1, with large contiguous patches resulting in larger Contiguity index values (**S1 Fig**).

#### Sub-regional scale

The distance from the city center (i.e. the Colosseum; lat.: 41.8902, long.: 12.4924) was also calculated. For Rome, this variable is a good proxy of decreasing disturbance along an urban-rural gradient [7,38], as suburban areas are richer in semi-natural habitats than the central areas (**Fig 1A**)

### Sampling protocol

In each study site, we sampled sphecids and tachinids using a set of 6 pan-traps, composed of yellow plastic cups (750 ml, Ø 12.5 cm, h 4.5 cm) filled with a solution of water and 2% biodegradable dish detergent. Pan-traps have been successfully used to collect flower-visiting insects in a wide range of habitats [10,11,36,48]. In the center of the study site, traps were placed on the ground (as the vegetation in the study areas was below 50 cm) at approximately 10 m apart, in two parallel lines of three pan-traps each. Field work was carried out every two weeks from early June to the end of September 2016, for a total of seven sampling rounds. Each sampling round, pan-traps were set out for 48 h. The material was sorted and identified at the Entomology laboratory of Sapienza, University of Rome (Italy). Specimens are preserved at the Museum of Zoology of Sapienza, University of Rome. No specific permissions were required for insect collection in the study area. The field work in this study did not involve endangered or protected species.

### Statistical analysis

All analyses were performed using R 3.2.2 [49]. Generalized linear models were used to explore how explanatory variables shape communities of sphecids and tachinids in the city of Rome. The response variables were the number of species (species richness), individuals (abundance), and evenness of each group per study site. Evenness was measured with Smith and Wilson’s index (E_var_) [50]. For species richness we used a quasi-poisson distribution whereas for abundance and evenness we used a normal distribution. To improve linearity, the abundance was log-transformed. Each model tested the effect of the explanatory variables at three spatial scales: 1) at the local scale, the distance of the sampling point from the street (log-transformed) and the coverage in buildings in a 50 m buffer; 2) at the landscape scale, the percentage of urban, and the area and Contiguity index of open green habitat; 3) at the sub-regional scale, the distance from the city center. The interaction between habitat area and Contiguity index was also tested. We expected that an higher Contiguity index (more contiguous habitat patches) may reduce the negative effect of habitat loss in small habitat areas, whereas, when the area is large, the effect of Contiguity index may be less important. Since the collinearity between variables was low (**S2 Table**), we used traditional hypothesis-testing based on P-values. Full models were simplified with a backward stepwise model selection procedure (P < 0.05) using the ‘stats’ package on R. Generalized linear models were fitted using the glm function in the ‘base’ package for R.

#### Species richness estimate and sampling effort

To account for the undetected species in each sampling site, abundance-based asymptotic estimators (Chao index and first-order Jackknife) were also calculated and used as response variables in the generalized linear models described above. The comparison of the results using the raw and estimated species richness provides a way to evaluate the appropriateness of our sampling effort. Additionally, species rarefaction curves were constructed to describe the species accumulation in relation to sampling effort (i.e. sampling rounds). Abundance-based estimates were calculated using the ‘specpool’ function of the ‘vegan’ package [51]. Accumulation curves were drawn using the ‘accumcomp’ function from the library ‘BiodiversityR’ [52].

## Results

Eight hundred sixteen predator and parasitoid insects were collected and identified to species level: 516 individuals (50 species) of sphecids (Ampulicidae, Sphecidae, Crabronidae), out of 380 species currently known from Italy [25], and 300 individuals (51 species) of tachinids (Tachinidae), out of 640 species currently known from Italy [44,53] (**S3 Table**). The most abundant species in each group were *Solierella compedita* (Piccioli) and *Cylindromyia pusilla* (Meigen), representing 29% and 25% of all the individual sphecids and tachinids, respectively. The Chao species richness estimator (corrected for unsampled species) indicated a total species richness of 79 ± 20 for sphecids and 85 ± 17 tachinids in the study area. The estimated rarefaction curves for each group showed a little decrease in the number of new species recorded at each round, higher for sphecids than tachinids (**S2 Fig**). However, results obtained using abundance-based asymptotic estimators (Chao index and first-order Jackknife) were similar to those obtained using observed species richness (**S4 Table**). Variables at all spatial scales (i.e. local, landscape and sub-regional) influenced species richness, abundance and evenness of both sphecids and tachinids (**Table 2**).

**Table 2:** Results from the generalized linear models testing the effect of the explanatory variables on: a) species richness, b) abundance, and c) evenness of sphecids and tachinids. Only significant results after a backward stepwise model selection procedure (P < 0.05) are reported.

		a) Species richness				b) Abundance				c) Evenness				Scale
		Est.	SE	t	P	Est.	SE	t	P	Est.	SE	t	P	
Sphecids	Distance from the street (log)	-	-	-	-	-	-	-	-	0.052	0.016	3.23	0.003	Local
	Buildings in 50 m	-	-	-	-	-	-	-	-	-	-	-	-	
	Percentage of urban	-	-	-	-	-0.019	0.008	-2.466	0.02	0.005	0.001	3.833	0.001	Landscape
	Habitat area	-	-	-	-	-0.165	0.043	-3.807	< 0.0001	0.035	0.006	5.434	< 0.0001	
	Habitat Contiguity index	-	-	-	-	-9.198	2.59	-3.552	0.001	1.723	0.393	4.386	< 0.0001	
	Habitat area:Contiguity index	-	-	-	-	0.284	0.088	3.217	0.003	-0.054	0.013	-4.049	< 0.0001	
	Distance from city center	0.352	0.157	2.239	0.032	1.164	0.485	2.4	0.023	-	-	-	-	Sub-regional
Tachinids	Distance from the street (log)	0.284	0.075	3.776	0.001	0.335	0.116	2.9	0.006	-	-	-	-	Local
	Buildings in 50 m	-0.003	0.001	-2.434	0.02	-	-	-	-	-	-	-	-	
	Percentage of urban	-	-	-	-	-	-	-	-	-	-	-	-	Landscape
	Habitat area	-	-	-	-	-	-	-	-	-	-	-	-	
	Habitat Contiguity index	-	-	-	-	-	-	-	-	0.92	0.197	4.677	< 0.0001	
	Habitat area:Contiguity index	-	-	-	-	-	-	-	-	-	-	-	-	
	Distance from city center	-	-	-	-	-	-	-	-	-	-	-	-	Sub-regional

### Local scale

**Fig 2:**
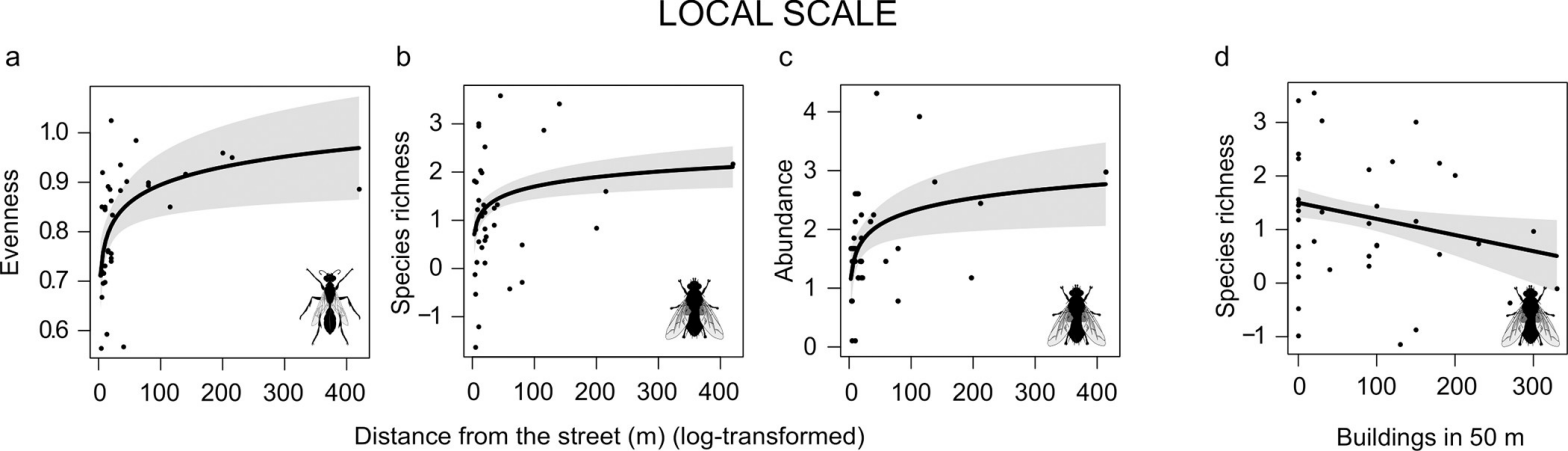
Effects of local scale variables. Plots showing the relationship between the distance from the street and: a) the evenness of sphecids, and b) the species richness and c) the abundance of tachinids. Panel **d** shows the effect of the coverage in buildings in a 50 m buffer on the species richness of tachinids. Only significant results were presented (P < 0.05). Plots include expected value (black line), and 95% confidence interval (gray shading).

At the local scale, the distance to the closest street increased evenness of sphecids, and species richness and abundance of tachinids (**Fig 2A, 2B and 2C**), while an increased coverage in buildings in a 50 m buffer negatively influenced species richness of tachinids (**Fig 2D**).

### Landscape scale

**Fig 3:**
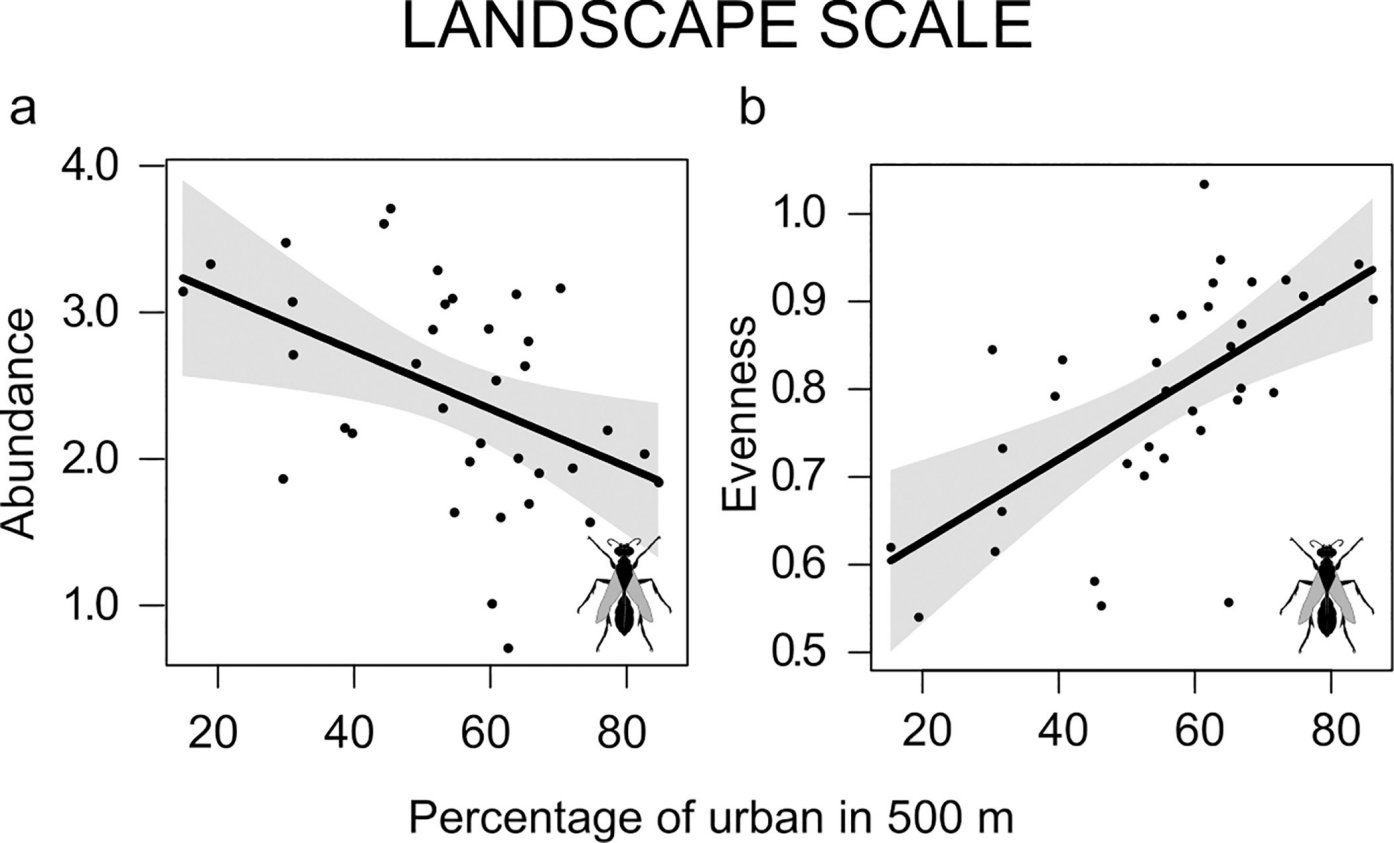
Effects of landscape scale variables: Percentage of urban. Plots showing the relationship between the percentage of urban in a 500 m radius buffer and a) the abundance and b) the evenness of sphecids. Only significant results were presented (P < 0.05). Plots include expected value (black line), and 95% confidence interval (gray shading).

**Fig 4:**
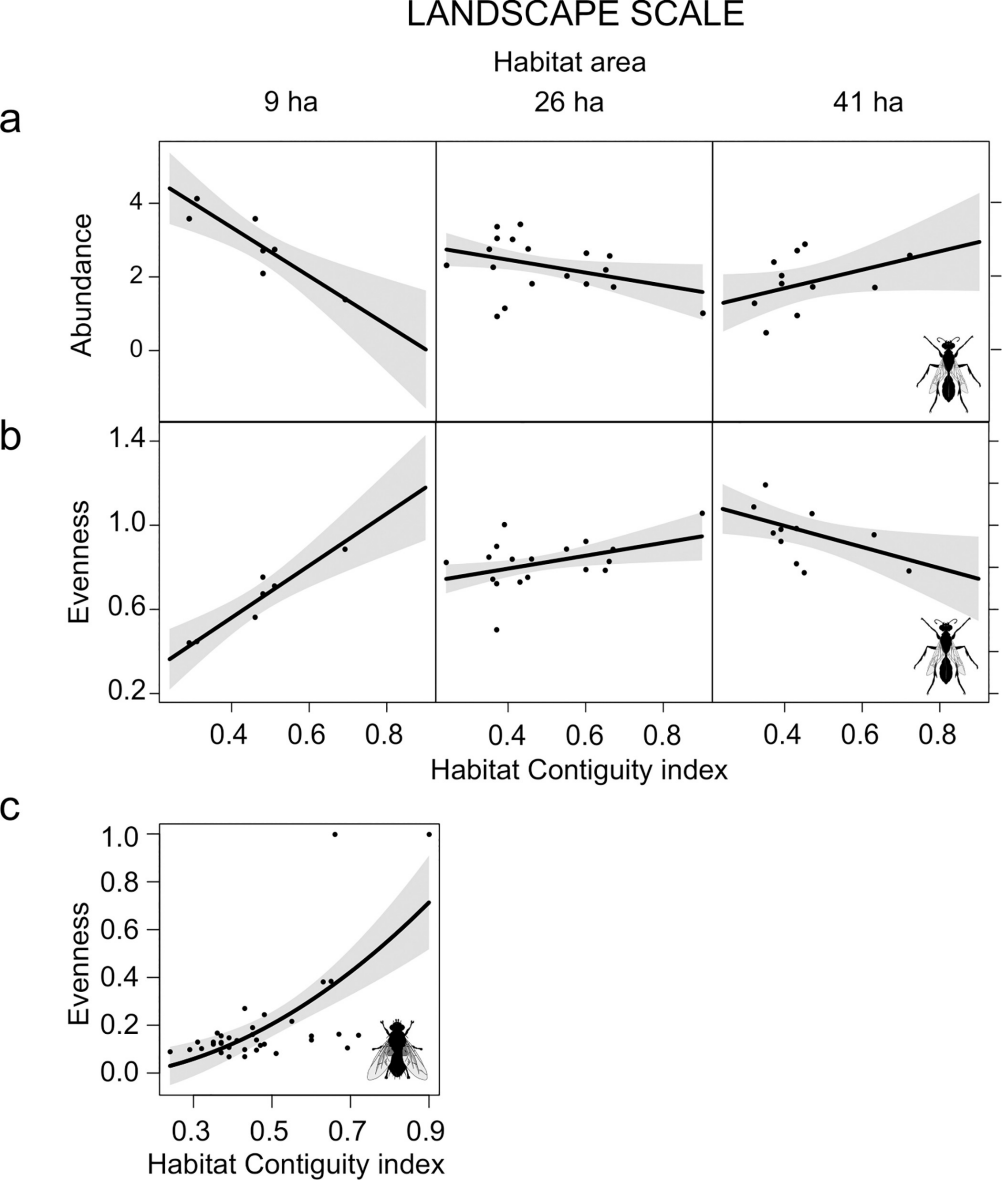
Effects of landscape scale variables: Habitat area and contiguity index. Plots showing the effects of the interaction of habitat area and habitat Contiguity index on a) the abundance and b) the evenness of sphecids, and c) the overall effect of habitat Contiguity index on the evenness of tachinids. Only significant results were presented (P < 0.05). Plots include expected value (black line), and 95% confidence interval (gray shading).

At the landscape scale, abundance of sphecids decreased, while evenness increased, with increasing percentage of urban area (**Fig 3A and 3B**). Also, the interaction between habitat area and Contiguity index influenced abundance and evenness of sphecids. When habitat area is small the Contiguity index reduced sphecid abundance, but increased evenness. As habitat area increased, the effect of the Contiguity index became neutral (at intermediate values of habitat area), while at high habitat area values, habitat contiguity increased sphecids abundance but decreased evenness (**Fig 4A and 4B**). Evenness of tachinids only depended on the Contiguity index, with increased evenness as habitat contiguity increased (**Fig 4C**).

### Sub-regional scale

**Fig 5:**
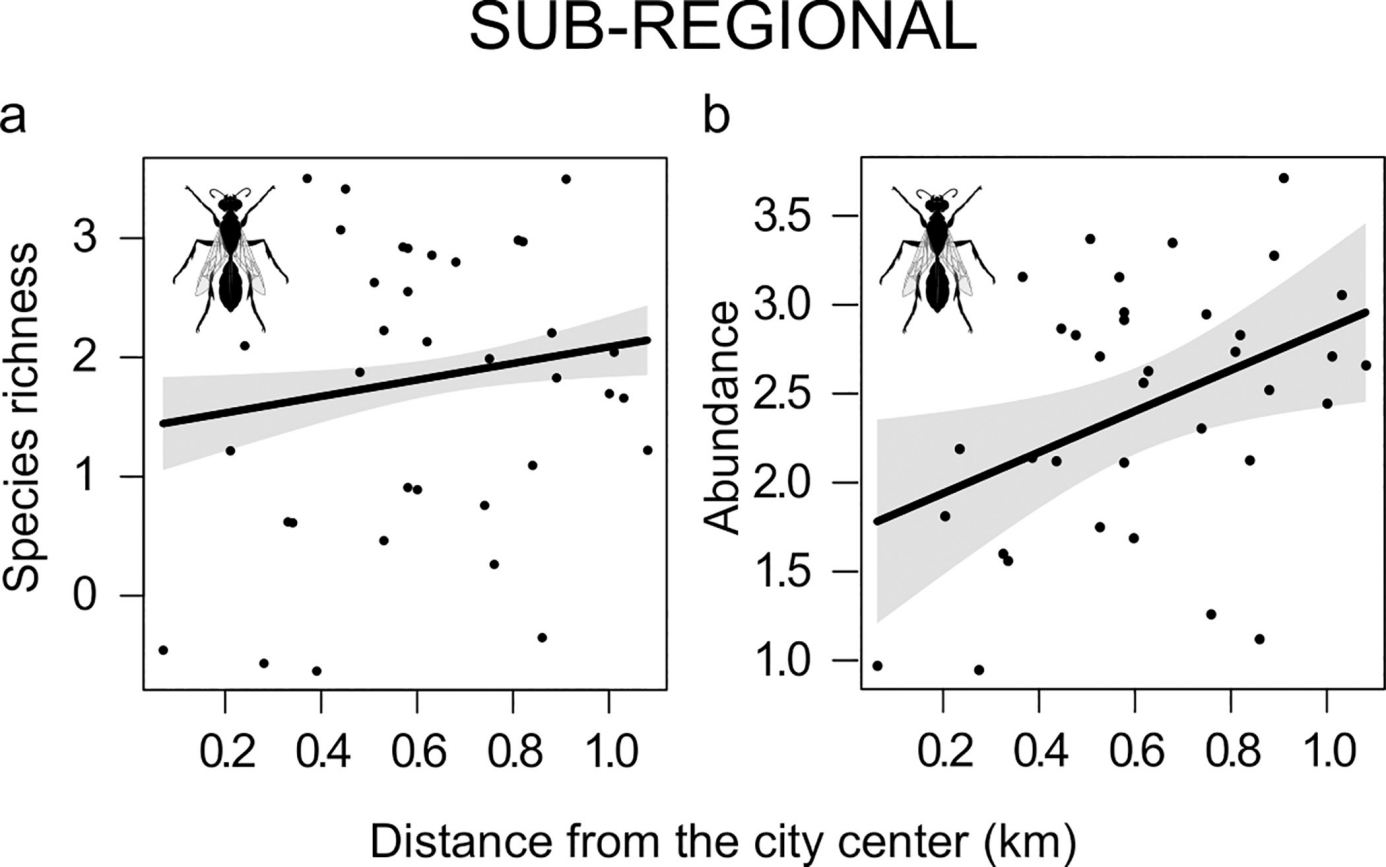
Effect of the sub-regional scale variable. Plots showing relationship between the distance from the city center and a) the species richness and b) the abundance of sphecids. Only significant results were presented (P < 0.05). Plots include expected value (black line), and 95% confidence interval (gray shading).

At the sub-regional scale, both species richness and abundance of sphecids were higher in sites distant from the city center (**Fig 5A and 5B**).

## Discussion

We investigated the effect of urban and open green habitat variables on species richness and evenness of predator and parasitoid insects. We found that urbanization at all spatial scales (i.e. local, landscape and sub-regional) influenced species richness or evenness of both groups, revealing complex responses of predator and parasitoid insects. Also, the effect of urbanization differed among groups and diversity measures. Studies exploring the effects of urbanization on flower-visiting species have also revealed mixed responses. Some species decline and become locally extinct in urban areas [2,18,20,21], whereas others remain common in highly disturbed habitats [3,4,9,10,13]. Several studies suggested that flower-visitors are affected by the availability and diversity of foraging and nesting resources rather than urbanization intensity itself [2,18,20–22,35,54].

### Local scale

Areas dominated by high-density buildings and close to streets were poor in tachinid species richness and abundance, while the proximity to street negatively affected sphecid evenness. Despite both groups are strong fliers [31,36], in urban areas the distance from street can affect their species richness and abundance (tachinids) or community evenness (sphecids). Tachinids are parasitoids and their diversity depend on the availability and abundance of hosts in the landscape [26]. Urban elements at local scale are likely perceived as barriers and negatively influence insect dispersal and location of potentially available hosts. Also, urbanization influences trophic interactions, favoring generalist species able to exploit multiple resources [17,18,30,33]. For sphecids we found that species evenness was lower in areas close to streets, probably because in these conditions the community composition was dominated by a few strong generalist (such as *Solierella compedita*) able to exploit resources in highly disturbed habitats.

### Landscape scale

The disturbance resulting from human activities at large spatial scale is one of the most important drivers of insect species diversity [8,18]. For example, urbanization negatively affect the diversity of bumble bees [2], bees and hoverflies [20,21]. On the other hand, some species can exploit the resources available in urban areas and are able to survive in highly disturbed habitats [4,8–10,13,48,54]. For example, nesting bees and wasps [3,4,10], as well as other generalist insect species [18,33], have been shown to benefit from urbanization. Here, we found that at the landscape scale the percentage of urban area decreased abundance and increased evenness of sphecids. This result indicates that highly urbanized areas support the same number of species present in areas rich in urban green, despite urbanization reduces the number of individuals and increased evenness. Marini et al. [55] found that habitat fragmentation (i.e. a reduction in semi-natural habitat area) increased pollinator evenness, due to the ability of highly mobile, generalist species to forage in disturbed landscapes. Similarly, sphecid community evenness may benefit from the novel resources available in highly urbanized landscapes. Many sphecid species often live in association with human settlements [56], and several of them have been previously showed to prefer anthropogenic habitats for nesting [34]. Additionally, among the sphecids collected, 12 species (28% of specimens collected) feed on spiders, which may be easier to locate on building walls than in semi-natural vegetation.

Urbanization also leads to habitat loss and fragmentation. The increase in habitat edges, together with the reduction in high quality habitat, usually reduce species diversity [12,16]. Surprisingly, species richness of both groups was not affected by overall habitat area. Habitat area is often identified as an important variable for insects in urban areas [7,30,57]. However, it may be of minor importance for highly mobile species belonging to higher trophic levels, because they can move across the landscape and are more affected by the availability and abundance of resources [2,15,20]. The habitat Contiguity index, on the other hand, influenced abundance and/or evenness of both groups. Overall Contiguity index increased tachinid evenness, whereas both abundance and evenness of sphecids depended on the interaction between area and Contiguity index of open green habitat. As habitat fragmentation involves a reduction in habitat compactness, it can reduce dispersal success and increase probability of regional extinction. More contiguous habitats (high Contiguity index) should support more stable communities, as in the case of tachinids. For sphecids, however, this trend only emerges when the overall habitat area is small. Marini et al. [55] found that pollinator communities inhabiting small and poorly connected habitat fragments were mainly composed of mobile and generalist species. Similarly, we found that in small habitat areas, the Contiguity index has a strong positive effect of on sphecid evenness, as an increased habitat compactness allows non-resident, mobile, generalist species to forage also in highly urbanized habitats. As habitat area increases, local populations have better availability of food resources and nesting sites [55]. Thus, also less generalist species are allowed to maintain viable population in contiguous habitat patches. The presence of rare species (e.g. singletons) lowers community evenness by increasing the differences in abundance between common and rare species [55].

### Sub-regional scale

The city of Rome is composed of large areas of non-agricultural green areas embedded in the urban landscape. These areas can play an important role as dispersal corridors, connecting areas of urban green with natural and semi-natural habitats surrounding the city [7,40]. Accordingly, species richness and abundance of sphecids was higher in suburban rather than central areas, probably because species nesting in wood and vegetation benefit from the proximity to large open areas surrounding the city.

## Conclusions

Compared to previous studies testing the effect of urbanization (e.g. [16,18,23,33]), we found little variation in species richness, abundance and evenness along the urbanization gradient. Several hypotheses may explain this pattern. First, it may be possible that the sites surveyed in Rome are already so intensively influenced by human activities that the most sensitive species are no longer present. Sattler et al. [58] found that insect diversity increased with age of urban settlement. The current insect fauna has probably been selected for its tolerance to habitat loss and fragmentation, being the result of the intensive anthropogenic alteration occurred in the area in the last centuries. Accordingly, highly specialized and rare species were rare, while an highly invasive wasp, *Chalybion bengalense* (Dahlbom) (Sphecidae), was recorded for the first time in the study area. Second, many insect species have been shown to be resilient to changes in land-use, except in cases of extreme habitat loss [7,9]. Moreover, Rome presents large unmanaged or irregularly managed green areas. These green areas can potentially provide foraging, nesting and overwintering habitats for both predators/parasitoids and their prey/hosts. Finally, environmental and structural variables often covary over the urbanization gradient [9], making it impossible to identify all explanatory variables driving diversity in urban areas. For example, ornamental flowers in private gardens can provide resources for flower-visiting insects, whose abundance can sometimes be enhanced in urban areas [20,23]. Although it may be difficult to measure presence and abundance of ornamental flowers around sampling areas, especially when sampling large areas, this variable may be a key factor in influencing flower-visitors in cities, and it may be useful to include it in further investigations.

This study highlights the importance of considering multi-scale variables to fully understand the effect of urbanization on high trophic levels. As predator and parasitoid insects require several feeding and nesting resources during their life cycle, their response to habitat fragmentation may be complex to predict. Dispersal and foraging of flying insects can occur at large spatial scales, especially in agricultural landscapes. However, urban elements, such as buildings and streets, can be perceived as obstacles, and limit insect movement also at smaller scales. Despite we found that the distance from the street was a good predictor of predator and parasitoid diversity, focusing conservation only on large habitat areas may not be the best way to maximize the conservation of flower-visiting insects in urban ecosystems. An approach that also considers the shape of habitat and complementarity of resources at the landscape scale may better improve the conservation of predators and parasitoids, including the ecological services they provide.

## Supporting information

S1 Fig Example of contiguity index in the study area. (DOCX)

S2 Fig Accumulation curves of sphecid and tachinid species richness against the number of sampling rounds. (DOCX)

S1 Table Selected sites and coordinates (WGS 84). (DOCX)

S2 Table Collinearity and multicollinearity between explanatory variables. (DOCX)

S3 Table Species lists, relative abundances and trophic behaviors of the larvae for the two predator and parasitoid groups in the study areas. (DOCX)

S4 Table Results from the generalized linear models testing the effect of the explanatory variables on abundance-based estimates of sphecids and tachinids: (a) Chao, and (b) first-order Jackknife.
(DOCX)

S1 Appendix Explanatory variables in each of the 36 selected sampling sites. (DOCX)
