# Supplementary material for: Impact of urbanization on predator and parasitoid insects at multiple spatial scales
Source: PLoS One. 2019 Apr 3;14(4):e0214068. doi: 10.1371/journal.pone.0214068 (PMC6447152; doi:10.1371/journal.pone.0214068)
Supplement: S1 Fig — (DOCX) [file pone.0214068.s001.docx]

**Supporting Information**

**Figure S1. Example of Contiguity index in the study area.** Contiguity index describes the geometric compactness of the habitat type in the landscape, and ranges from 0 to 1, with large contiguous patches resulting in larger Contiguity index values.

**
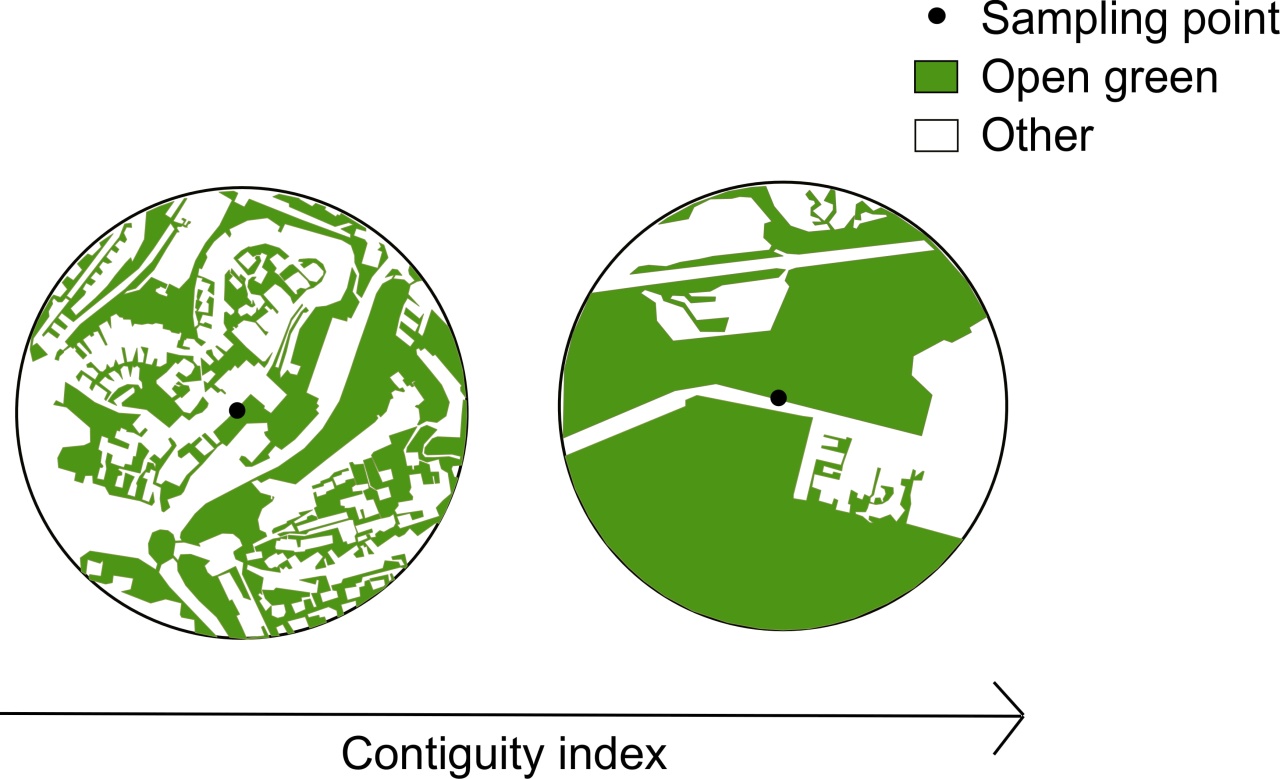
**
