# Supplementary material for: Impact of urbanization on predator and parasitoid insects at multiple spatial scales
Source: PLoS One. 2019 Apr 3;14(4):e0214068. doi: 10.1371/journal.pone.0214068 (PMC6447152; doi:10.1371/journal.pone.0214068)

**Supporting Information**

**Figure S2. Accumulation curves of sphecid and tachinid species richness against the number of sampling rounds.** The vertical lines in the accumulation curves represent the 95% CI. Rarefaction curves are based on 1000 randomizations.


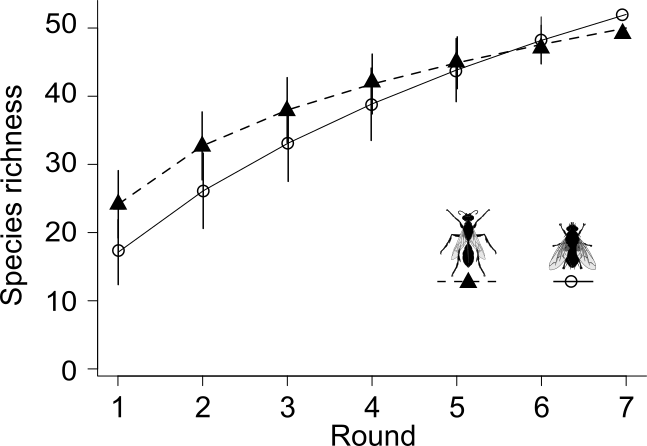

Supplement: S2 Fig — (DOCX) [file pone.0214068.s002.docx]
