# Supplementary material for: Impact of urbanization on predator and parasitoid insects at multiple spatial scales
Source: PLoS One. 2019 Apr 3;14(4):e0214068. doi: 10.1371/journal.pone.0214068 (PMC6447152; doi:10.1371/journal.pone.0214068)
Supplement: S1 Table — (DOCX) [file pone.0214068.s003.docx]

**Supporting Information**

**S1 Table.** **Selected sites and coordinates (WGS 84).**

| Site ID | lat | long | City | Country |
| --- | --- | --- | --- | --- |
| 57 | 12.5026 | 41.9736 | Rome | Italy |
| 75 | 12.4435 | 41.9547 | Rome | Italy |
| 93 | 12.3838 | 41.9440 | Rome | Italy |
| 122 | 12.4564 | 41.9369 | Rome | Italy |
| 125 | 12.4916 | 41.9390 | Rome | Italy |
| 127 | 12.5168 | 41.9386 | Rome | Italy |
| 130 | 12.5536 | 41.9407 | Rome | Italy |
| 132 | 12.5771 | 41.9392 | Rome | Italy |
| 149 | 12.5048 | 41.9290 | Rome | Italy |
| 151 | 12.5285 | 41.9294 | Rome | Italy |
| 157 | 12.6004 | 41.9317 | Rome | Italy |
| 164 | 12.4082 | 41.9177 | Rome | Italy |
| 166 | 12.4330 | 41.9170 | Rome | Italy |
| 173 | 12.5179 | 41.9192 | Rome | Italy |
| 188 | 12.4201 | 41.9074 | Rome | Italy |
| 193 | 12.4792 | 41.9102 | Rome | Italy |
| 198 | 12.5412 | 41.9125 | Rome | Italy |
| 220 | 12.5275 | 41.9016 | Rome | Italy |
| 234 | 12.4238 | 41.8936 | Rome | Italy |
| 237 | 12.4584 | 41.8921 | Rome | Italy |
| 246 | 12.5676 | 41.8925 | Rome | Italy |
| 254 | 12.3869 | 41.8843 | Rome | Italy |
| 263 | 12.4944 | 41.8839 | Rome | Italy |
| 287 | 12.5083 | 41.8726 | Rome | Italy |
| 290 | 12.5427 | 41.8758 | Rome | Italy |
| 307 | 12.4718 | 41.8675 | Rome | Italy |
| 332 | 12.4970 | 41.8582 | Rome | Italy |
| 339 | 12.5797 | 41.8579 | Rome | Italy |
| 353 | 12.4710 | 41.8457 | Rome | Italy |
| 373 | 12.4351 | 41.8384 | Rome | Italy |
| 379 | 12.5129 | 41.8410 | Rome | Italy |
| 400 | 12.4839 | 41.8293 | Rome | Italy |
| 407 | 12.5689 | 41.8318 | Rome | Italy |
| 417 | 12.4071 | 41.8169 | Rome | Italy |
| 420 | 12.4502 | 41.8208 | Rome | Italy |
| 442 | 12.4371 | 41.8100 | Rome | Italy |
