# Supplementary material for: Impact of urbanization on predator and parasitoid insects at multiple spatial scales
Source: PLoS One. 2019 Apr 3;14(4):e0214068. doi: 10.1371/journal.pone.0214068 (PMC6447152; doi:10.1371/journal.pone.0214068)
Supplement: S2 Table — (DOCX) [file pone.0214068.s004.docx]

**Supporting Information**

**S2 Table.** **Collinearity and multicollinearity between explanatory variables**. a) Pearson’s correlation indices, and b) Variance Inflation Factors

|  | **a) Pearson’s correlation** | | | | | | **b) VIF** |
| --- | --- | --- | --- | --- | --- | --- | --- |
|  | Distance from city center | Distance from the street | Buildings in 50 m | Percentage of urban | Habitat area | Habitat Contiguity index |  |
| Distance from city center | 1.00 |  |  |  |  |  | 1.07 |
| Distance from the street | -0.05 | 1.00 |  |  |  |  | 1.53 |
| Buildings in 50 m | -0.11 | -0.11 | 1.00 |  |  |  | 1.42 |
| Percentage of urban | -0.11 | -0.34 | 0.52 | 1.00 |  |  | 2.20 |
| Habitat area | 0.24 | -0.21 | -0.28 | -0.47 | 1.00 |  | 1.67 |
| Habitat Contiguity index | 0.10 | 0.24 | -0.13 | -0.02 | -0.05 | 1.00 | 1.07 |
