# Supplementary material for: Impact of urbanization on predator and parasitoid insects at multiple spatial scales
Source: PLoS One. 2019 Apr 3;14(4):e0214068. doi: 10.1371/journal.pone.0214068 (PMC6447152; doi:10.1371/journal.pone.0214068)
Supplement: S3 Table — (DOCX) [file pone.0214068.s005.docx]

**Supporting Information**

**S3 Table.** **Species lists, relative abundances and trophic behaviors of the larvae for the two predator and parasitoid groups in the study areas**. For sphecids the nesting site was also provided. Although the aim of this study was not to provide a complete checklist of the study area, previous checklists of Rome are still largely incomplete, and 38 species of sphecids and 11 of tachinids have not been reported for the area before. Also, the invasive sphecid wasp *Chalybion bengalense* (Dahlbom) was recorded for the first time in the Lazio region.

|  |  | **Sphecids** |  |  |
| --- | --- | --- | --- | --- |
| **Species** | **Tot** | **Trophic behavior of larvae** | **Nesting site** | **Previous reports for the area** |
| *Astata apostata* Mercet, 1910 | 19 | Predator of Hemiptera | ground |  |
| *Astata costae* A. Costa, 1867 | 6 | Predator of Hemiptera | ground |  |
| *Astata kashmirensis* Nurse, 1909 | 1 | Predator of Hemiptera | ground | Zapparoli 1997 |
| *Astata minor* Kohl, 1885 | 1 | Predator of Hemiptera | ground |  |
| *Bembecinus tridens* (Fabricius 1781) | 6 | Predator of Homoptera | ground | Zapparoli 1997 |
| *Bembix oculata* Panzer, 1801 | 2 | Predator of Diptera | ground |  |
| *Cerceris arenaria* (Linnaeus, 1758) | 1 | Predator of Coleoptera | ground | Zapparoli 1997 |
| *Cerceris sabulosa* (Panzer 1799) | 5 | Predator of Hymenoptera | ground | Zapparoli 1997 |
| *Chalybion bengalense* (Dahlbom, 1845) | 1 | Predator of Araneae | mud nest |  |
| *Crossocerus* cf. *wesmaeli* (Shuckard, 1837) | 7 | Predator of Diptera/Homoptera | NA | Zapparoli 1997 |
| *Diodontus minutus* (Fabricius, 1793) | 12 | Predator of Homoptera (aphids) | ground |  |
| *Dolichurus corniculus* (Spinola 1808) | 11 | Predator of Blattodea | ground |  |
| *Dolichurus haemorrhous*A. Costa 1886 | 4 | Predator of Blattodea | ground |  |
| *Dryudella tricolor* (Vander Linden, 1829) | 5 | Predator of Hemiptera | ground |  |
| *Harpactus laevis* (Latreille 1792) | 1 | Predator of Homoptera | ground |  |
| *Harpactus pulchellus* (A. Costa, 1859) | 1 | Predator of Homoptera | ground |  |
| *Harpactus quadrisignatus* (Palma, 1869) | 34 | Predator of Homoptera | ground |  |
| *Lestica clypeata* (Schreber, 1759) | 4 | Predator of Lepidoptera | wood, stems | Zapparoli 1997 |
| *Lindenius pygmaeus* (Rossi, 1794) | 1 | Predator of Hymenoptera | ground | Zapparoli 1997 |
| *Miscophus eatoni* E. Saunders 1903 | 6 | Predator of Araneae | ground |  |
| *Nitela borealis* Valkeila 1974 | 2 | Predator of Psocoptera | wood, stems |  |
| *Nysson maculosus* (Gmelin 1790) | 1 | Cleptoparasite | ground |  |
| *Nysson tridens* Gerstaecker, 1867 | 1 | Cleptoparasite | ground |  |
| *Nysson variabilis* Chevrier, 1867 | 7 | Cleptoparasite | NA |  |
| *Passaloecus eremita* Kohl, 1893 | 2 | Predator of Homoptera (aphids) | wood, stems |  |
| *Passaloecus singularis* Dahlbom 1844 | 12 | Predator of Homoptera (aphids) | wood, stems |  |
| *Pemphredon inornata* Say 1824 | 2 | Predator of Homoptera (aphids) | wood, stems |  |
| *Pemphredon lethifer* (Shuckard 1837) | 7 | Predator of Homoptera (aphids) | wood, stems |  |
| *Philanthus triangulum*(Fabricius, 1775) | 1 | Predator of Hymenoptera | ground | Zapparoli 1997 |
| *Pison atrum* (Spinola 1808) | 2 | Predator of Araneae | wood, stems |  |
| *Sceliphron caementarium* (Drury 1770) | 1 | Predator of Araneae | mud nest |  |
| *Solierella compedita* (Piccioli 1969) | 148 | Predator of Hemiptera | wood, stems, ground |  |
| *Sphex funerarius* Gussakovskij 1934 | 1 | Predator of Orthoptera | ground |  |
| *Spilomena mocsaryi* Kohl 1898 | 6 | NA | NA |  |
| *Stigmus solskyi* A. Morawitz 1864 | 2 | Predator of Homoptera (aphids) | wood, stems |  |
| *Tachysphex fulvitarsis* A. Costa 1867 | 10 | Predator of Orthoptera | ground |  |
| *Tachysphex incertus* Radoszkowski 1877 | 4 | Predator of Orthoptera | ground |  |
| *Tachysphex mediterraneus* Kohl 1883 | 1 | Predator of Orthoptera | ground |  |
| *Tachysphex nitidior* Beaumont 1940 | 10 | Predator of Orthoptera | ground | Zapparoli 1997 |
| *Tachysphex obscuripennis* (Schenck, 1857) | 1 | Predator of Blattodea | ground | Zapparoli 1997 |
| *Tachysphex tarsinus* (Lepeletier 1845) | 30 | Predator of Orthoptera | ground |  |
| *Tracheliodes quinquenotatus* (Jurine, 1807) | 3 | Predator of Hymenoptera (ants) | ground | Zapparoli 1997 |
| *Trypoxylon attenuatum* F. Smith 1851 | 30 | Predator of Araneae | wood, stems | Zapparoli 1997 |
| *Trypoxylon clavicerum*Lepeletier & Serville 1828 | 6 | Predator of Araneae | wood, stems |  |
| *Trypoxylon deceptorium* Antropov 1991 | 15 | Predator of Araneae | wood, stems |  |
| *Trypoxylon figulus* (Linnaeus 1758) | 1 | Predator of Araneae | wood, ground |  |
| *Trypoxylon kolazyi* Kohl 1893 | 5 | Predator of Araneae | wood, ground |  |
| *Trypoxylon medium* Beaumont 1945 | 16 | Predator of Araneae | wood, stems |  |
| *Trypoxylon minus* Beaumont 1945 | 60 | Predator of Araneae | wood, stems |  |
| *Trypoxylon scutatum* Chevrier 1867 | 1 | Predator of Araneae | wood, ground |  |
| **Total of species** | **50** |  |  |  |
| **Total of specimens** | **516** |  |  |  |

| **Tachinids** | | | |
| --- | --- | --- | --- |
|  | | | |
| **Species** | **Tot** | **Trophic behavior of larvae** | **Previous reports for the area** |
| *Actia pilipennis* (Fallén, 1810) | 1 | parasitoid | Cerretti 2001 |
| *Aplomya confinis* (Fallén, 1820) | 1 | parasitoid | Cerretti 2001; Zapparoli 1997 |
| *Atylomyia loewii* Brauer, 1898 | 1 | parasitoid | Cerretti 2001 |
| *Besseria reflexa* Robineau-Desvoidy, 1830 | 5 | parasitoid |  |
| *Catharosia pygmaea* (Fallén, 1815) | 1 | parasitoid | Cerretti 2001 |
| *Chaetoria stylata* Becker, 1908 | 1 | parasitoid | Cerretti 2001 |
| *Clairvillia pninae* Kugler, 1971 | 1 | parasitoid | Cerretti 2001 |
| *Clemelis pullata* (Meigen, 1824) | 1 | parasitoid |  |
| *Cylindromyia bicolor* (Olivier, 1812) | 1 | parasitoid | Cerretti 2001; Zapparoli 1997 |
| *Cylindromyia hermonensis* Kugler, 1974 | 2 | parasitoid | Cerretti 2001 |
| *Cylindromyia intermedia* (Meigen, 1824) | 6 | parasitoid | Cerretti 2001 |
| *Cylindromyia pusilla* (Meigen, 1824) | 74 | parasitoid | Cerretti 2001; Zapparoli 1997 |
| *Cyrtophleba ruricola* (Meigen, 1824) | 1 | parasitoid | Cerretti 2001 |
| *Dinera grisescens* (Fallén, 1817) | 1 | parasitoid | Cerretti 2001 |
| *Drino atropivora* (Robineau-Desvoidy, 1830) | 2 | parasitoid | Zapparoli 1997 |
| *Ectophasia crassipennis* (Fabricius, 1794) | 1 | parasitoid | Cerretti 2001; Zapparoli 1997 |
| *Erynniopsis antennata* (Rondani, 1861) | 1 | parasitoid |  |
| *Exorista civilis* (Róndani, 1859) | 1 | parasitoid |  |
| *Exorista larvarum* (Linnaeus, 1758) | 1 | parasitoid | Cerretti 2001; Zapparoli 1997 |
| *Exorista xanthaspis* (Wiedemann, 1830) | 2 | parasitoid | Zapparoli 1997 |
| *Gastrolepta anthracina* (Meigen, 1826) | 12 | parasitoid | Cerretti 2001 |
| *Gymnosoma nitens* Meigen, 1824 | 1 | parasitoid | Cerretti 2001 |
| *Hyperaea femoralis (Meigen, 1824)* | 1 | parasitoid | Cerretti 2001 |
| *Leucostoma tetraptera* (Meigen, 1824) | 8 | parasitoid |  |
| *Linnaemya frater* (Rondani, 1859) | 2 | parasitoid | Cerretti 2001 |
| *Medina melania* (Meigen, 1824) | 2 | parasitoid |  |
| *Medina separata* (Meigen, 1824) | 8 | parasitoid | Cerretti 2001 |
| *Meigenia mutabilis* (Fallén, 1810) | 1 | parasitoid | Cerretti 2001 |
| *Meigenia mutabilis* group | 4 | parasitoid | Cerretti 2001 |
| *Mintho rufiventris* (Fallén, 1817) | 10 | parasitoid | Zapparoli 1997 |
| *Nemorilla maculosa* (Meigen, 1824) | 3 | parasitoid |  |
| *Nemoraea pellucida* (Meigen, 1824) | 3 | parasitoid | Cerretti 2001; Zapparoli 1997 |
| *Pales pavida* (Meigen, 1824) | 3 | parasitoid | Cerretti 2001 |
| *Pales processioneae* (Ratzeburg, 1840) | 2 | parasitoid | Cerretti 2001 |
| *Peleteria rubescens* (Robineau-Desvoidy, 1830) | 1 | parasitoid | Cerretti 2001; Zapparoli 1997 |
| *Peleteria ruficornis* (Macquart, 1835) | 1 | parasitoid | Cerretti 2001 |
| *Peribaea tibialis* (Robineau-Desvoidy, 1851) | 2 | parasitoid | Cerretti 2001 |
| *Phania funesta* (Meigen, 1824) | 4 | parasitoid | Cerretti 2001 |
| *Phryxe vulgaris* (Fallén, 1810) | 1 | parasitoid | Cerretti 2001; Zapparoli 1997 |
| *Pseudogonia rufifrons* (Wiedemann, 1830) | 2 | parasitoid | Cerretti 2001; Zapparoli 1997 |
| *Pseudoperichaeta nigrolineata* (Walker, 1853) | 2 | parasitoid | Cerretti 2001 |
| *Pseudoperichaeta palesioidea* (Robineau-Desvoidy, 1830) | 1 | parasitoid |  |
| *Stomina caliendrata* (Rondani, 1862) | 55 | parasitoid | Zapparoli 1997 |
| *Stomina tachinoides* (Fallén, 1817) | 1 | parasitoid |  |
| *Tachina praeceps* (Meigen, 1824) | 1 | parasitoid | Zapparoli 1997 |
| *Thecocarcelia trichops* Herting, 1967 | 1 | parasitoid |  |
| *Thelaira nigripes* (Fabricius, 1794) | 1 | parasitoid | Zapparoli 1997 |
| *Thelyconychia solivaga* (Rondani, 1861) | 29 | parasitoid |  |
| *Triarthria setipennis* (Fallén, 1810) | 3 | parasitoid | Cerretti 2001; Zapparoli 1997 |
| *Trichopoda pennipes* (Fabricius, 1781) | 2 | parasitoid | Zapparoli 1997 |
| *Voria ruralis* (Fallén, 1810) | 26 | parasitoid | Cerretti 2001; Zapparoli 1997 |
| *Zeuxia aberrans* (Loew, 1847) | 2 | parasitoid | Cerretti 2001 |
| **Total of species** | **51** |  |  |
| **Total specimens** | **300** |  |  |

**Literature used for species identification**

Bitsch J, Barbier Y, Gayubo SF, Schmidt K, Ohl M (1997) Faune de France. France et régions limitrophes. 82. Hyménoptères Sphecidae d'Europe occidentale. Volume 2 Fédération Française des Sociétés de Sciences Naturelles, Paris. 429 pp.

Bitsch J, Dollfuss H, Boucek Z, Schmidt K, Schmid-Egger Ch, Gayubo SF, Antropov AV, Barbier Y (2001) Hyménoptères Sphecidae d'Europe Occidentale. Volume 3. Faune de France 86. Fédération française des Sociétés de Sciences Naturelles, Paris. 459 pp.

Bitsch J, Leclercq J (1993) Faune de France. France et régions limitrophes. 79. Hyménoptères Sphecidae d'Europe occidentale. Volume 1. Généralités – Crabroninae. Fédération Française des Sociétés de Sciences Naturelles, Paris. 325 pp.

Cerretti P (2010) I tachinidi della fauna italiana (Diptera Tachinidae), con chiave interattiva dei generi ovest paleartici, Vol. I & II, CD-rom (in Italian with English interactive key). Cierre Edizioni, Verona, Italy.

Cerretti P, Tschorsnig H-P, Lopresti M, Di Giovanni F (2012) MOSCHweb: a matrix-based interactive key to the genera of the Palaearctic Tachinidae (Insecta, Diptera). Zookeys 205:5–18.

Pagliano G, Negrisolo E (2005) Fauna d’Italia. Hymenoptera Sphecidae. Edizioni Calderini, Bologna. 559 pp.
