# Supplementary material for: Impact of urbanization on predator and parasitoid insects at multiple spatial scales
Source: PLoS One. 2019 Apr 3;14(4):e0214068. doi: 10.1371/journal.pone.0214068 (PMC6447152; doi:10.1371/journal.pone.0214068)
Supplement: S4 Table — Results from the generalized linear models testing the effect of the explanatory variables on abundance-based estimates of sphecids and tachinids: (a) Chao, and (b) first-order Jackknife. (DOCX) [file pone.0214068.s006.docx]

**Supporting Information**

**S4 Table. Results from the generalized linear models testing the effect of the explanatory variables on abundance-based estimates of sphecids and tachinids: (a) Chao, and (b) first-order Jackknife.** For both estimates we used a normal distribution. Only significant results after a backward stepwise model selection procedure (P < 0.05) are reported. Abundance-based estimates were calculated using the ‘specpool’ function of the ‘vegan’ package on R.

|  | | a) Chao | | | | b) Jackknife-1 | | | | Scale |
| --- | --- | --- | --- | --- | --- | --- | --- | --- | --- | --- |
|  |  | Estimate | SE | t | P | Estimate | SE | t | P |  |
| Sphecids | Distance from the street | - | - | - | - | - | - | - | - | Local |
|  | Buildings in 50 m | - | - | - | - | - | - | - | - |  |
|  | Percentage of urban | - | - | - | - | - | - | - | - |  |
|  | Habitat area | - | - | - | - | - | - | - | - |  |
|  | Habitat Contiguity index | - | - | - | - | - | - | - | - | Landscape |
|  | Habitat area* Contiguity index | - | - | - | - | - | - | - | - |  |
|  | Distance from city center | 14.472 | 5.912 | 2.448 | 0.020 | 7.372 | 2.878 | 2.562 | 0.015 | Sub-regional |
| Tachinids | Distance from the street | 2.542 | 1.096 | 2.320 | 0.027 | -0.019 | 0.007 | -2.519 | 0.017 | Local |
|  | Buildings in 50 m | -0.034 | 0.014 | -2.440 | 0.021 | 1.785 | 0.583 | 3.062 | 0.005 |  |
|  | Percentage of urban | - | - | - | - | - | - | - | - |  |
|  | Habitat area | - | - | - | - | - | - | - | - |  |
|  | Habitat Contiguity index | - | - | - | - | - | - | - | - | Landscape |
|  | Habitat area* Contiguity index | - | - | - | - | - | - | - | - |  |
|  | Distance from city center | - | - | - | - | - | - | - | - | Sub-regional |
