# Supplementary material for: Impact of urbanization on predator and parasitoid insects at multiple spatial scales
Source: PLoS One. 2019 Apr 3;14(4):e0214068. doi: 10.1371/journal.pone.0214068 (PMC6447152; doi:10.1371/journal.pone.0214068)
Supplement: S1 Appendix — (DOCX) [file pone.0214068.s007.docx]

**S1 Appendix**

**Explanatory variables in each of the 36 selected sampling sites**

| **Site ID** | **Distance from city center (km)** | **Distance from the street (m)** | **Buildings in 50 m** | **Percentage of urban** | **Habitat area** | **Habitat Contiguity index** |
| --- | --- | --- | --- | --- | --- | --- |
| **57** | 8.4 | 10 | 0 | 39.4 | 41.4 | 0.6 |
| **75** | 8.2 | 420 | 0 | 19.4 | 18.6 | 0.7 |
| **93** | 10.8 | 10 | 300 | 68.3 | 24.6 | 0.6 |
| **122** | 6 | 15 | 90 | 62.6 | 3.7 | 0.5 |
| **125** | 5.3 | 5 | 0 | 40.5 | 31.5 | 0.9 |
| **127** | 5.7 | 8 | 100 | 53.2 | 34.8 | 0.4 |
| **130** | 7.4 | 80 | 0 | 65.2 | 19.2 | 0.7 |
| **132** | 8.9 | 20 | 20 | 60.8 | 30.8 | 0.6 |
| **149** | 4.4 | 215 | 0 | 15.3 | 7.9 | 0.5 |
| **151** | 5.3 | 20 | 30 | 66.2 | 20.5 | 0.5 |
| **157** | 10.1 | 45 | 20 | 73.3 | 19.8 | 0.6 |
| **164** | 7.6 | 20 | 180 | 61.3 | 24.9 | 0.4 |
| **166** | 5.8 | 7 | 0 | 30.6 | 28.0 | 0.4 |
| **173** | 3.9 | 15 | 230 | 86.1 | 10.9 | 0.7 |
| **188** | 6.3 | 3 | 200 | 78.5 | 8.1 | 0.3 |
| **193** | 2.4 | 10 | 100 | 61.9 | 23.4 | 0.4 |
| **198** | 4.8 | 10 | 130 | 66.7 | 26.3 | 0.7 |
| **220** | 3.4 | 200 | 270 | 66.8 | 26.1 | 0.5 |
| **234** | 5.8 | 15 | 0 | 59.6 | 29.0 | 0.4 |
| **237** | 2.8 | 6 | 90 | 30.2 | 41.3 | 0.4 |
| **246** | 6.2 | 80 | 150 | 84.0 | 9.0 | 0.5 |
| **254** | 8.8 | 20 | 40 | 31.7 | 34.8 | 0.3 |
| **263** | 0.7 | 140 | 30 | 55.7 | 34.9 | 0.4 |
| **287** | 2.1 | 35 | 100 | 54.0 | 34.4 | 0.5 |
| **290** | 4.5 | 20 | 180 | 55.4 | 17.2 | 0.5 |
| **307** | 3.3 | 22 | 330 | 75.9 | 13.8 | 0.3 |
| **332** | 3.7 | 5 | 0 | 71.5 | 18.8 | 0.4 |
| **339** | 8.1 | 115 | 120 | 50.0 | 39.2 | 0.7 |
| **353** | 5.1 | 13 | 0 | 46.2 | 33.8 | 0.5 |
| **373** | 7.5 | 10 | 150 | 52.5 | 34.8 | 0.4 |
| **379** | 5.8 | 35 | 0 | 54.3 | 26.7 | 0.4 |
| **400** | 6.8 | 40 | 90 | 64.9 | 18.8 | 0.4 |
| **407** | 9.1 | 4 | 90 | 45.2 | 43.0 | 0.4 |
| **417** | 10.3 | 5 | 0 | 31.6 | 53.8 | 0.4 |
| **420** | 8.6 | 60 | 0 | 63.7 | 27.3 | 0.4 |
| **442** | 10 | 18 | 150 | 58.0 | 32.7 | 0.2 |
